# Aggregation favors singlet formation in TES-ADT triplet annihilator for photon upconversion

**DOI:** 10.1039/d5sc07013a

**Published:** 2026-01-28

**Authors:** Justas Lekavičius, Edvinas Radiunas, Gediminas Kreiza, Augustina Jozeliūnaitė, Edvinas Orentas, Karolis Kazlauskas

**Affiliations:** a Institute of Photonics and Nanotechnology, Faculty of Physics, Vilnius University Saulėtekio av. 3 LT-10257 Vilnius Lithuania karolis.kazlauskas@ff.vu.lt; b Institute of Chemistry, Faculty of Chemistry and Geosciences, Vilnius University Naugarduko 24 LT-03225 Vilnius Lithuania

## Abstract

Triplet–triplet annihilation (TTA)-mediated photon upconversion (UC) offers a promising route for transforming low-energy photons into higher-energy ones under low-power, incoherent excitation, with applications in photovoltaics, bioimaging, 3D printing, *etc.* However, a central constraint on UC efficiency is the limited spin-statistical factor (*f*), which dictates the yield of singlet state formation and is especially challenging in the desirable far-red/NIR spectral range. Here, we explore a new approach of tuning the annihilator's *f* factor through controlled aggregation. The study covers a systematic investigation of triethylsilyl-substituted anthradithiophene (TES-ADT) annihilator solutions across a range of concentrations, complemented by studies on a neat TES-ADT film and density functional theory (DFT) calculations. We report a remarkable 3-fold enhancement in singlet yield, boosting the *f* value from *ca.* 20% to an impressive *ca.* 60% upon increasing annihilator concentration, which is shown to be directly linked to annihilator aggregation. DFT calculations further suggest that dimerization-induced shifts in energy levels and the accessibility of higher-energy triplet states (up to T_6_) facilitate spin-conversion processes. Our findings unveil aggregation-enhanced singlet generation *via* TTA in TES-ADT, providing a valuable insight for designing more efficient UC systems by tailoring energy landscapes through molecular packing.

## Abstract 2

Aggregation of TES-ADT annihilator reshapes the energy landscape and enables access to higher-energy triplet states *via* TTA, thereby boosting the spin-statistical factor (*f*) from *ca.* 20% to 60% in far-red/NIR photon upconversion.

## Graphical abstract

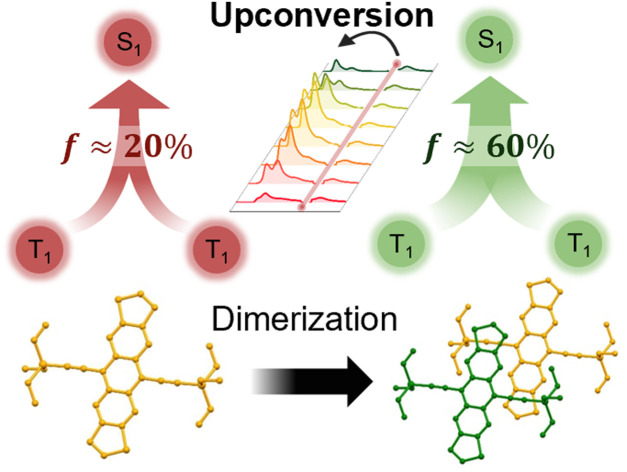

## Introduction

**Fig. 1:**
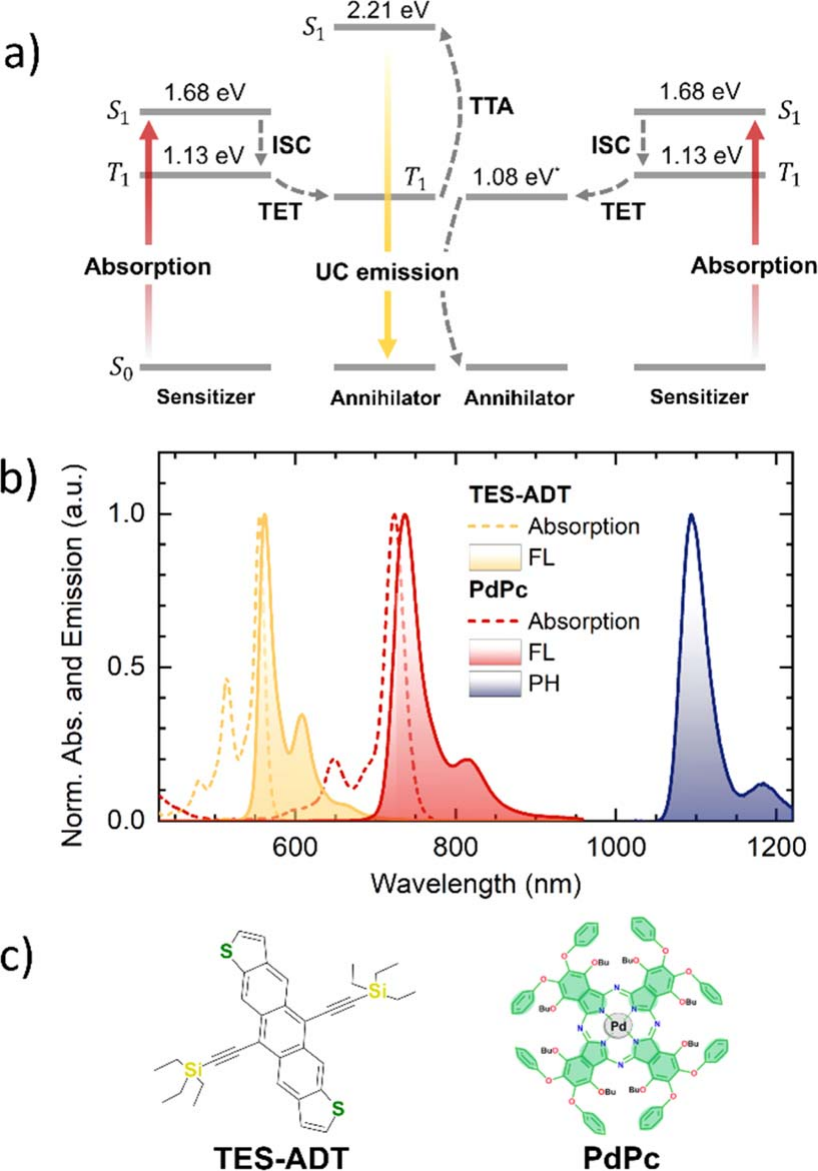
(a) Jablonski diagram of a TTA-UC system (TES-ADT:PdPc), showing key processes: ISC – intersystem crossing, TET – triplet energy transfer, and TTA – triplet–triplet annihilation. Annihilator and sensitizer energy levels were evaluated from the maxima of their FL and PH emission spectra. *T_1_ of TES-ADT was reported elsewhere.^28^ (b) Normalized absorption and emission spectra (FL – fluorescence, PH – phosphorescence) of 10 µM TES-ADT and PdPc in toluene. The sensitizer's PH spectrum was measured at 30 K from a 0.1 wt% film of PdPc in a polystyrene matrix. (c) Chemical structures of TES-ADT annihilator and PdPc sensitizer.

Sensitized triplet–triplet annihilation (TTA) within organic systems comprising tailored sensitizer and annihilator species can generate a noticeable fraction of high-energy singlets, a phenomenon known as photon upconversion (UC).^1^ In this process, the triplets are supplied to the annihilator *via* Dexter-type energy transfer mechanism from an energetically compatible sensitizer. The sensitizer initially absorbs lower-energy photons, populating its singlet state before undergoing intersystem crossing (ISC) to its triplet manifold (Fig. 1a). Notably, optimized TTA-UC systems can sustain this energy conversion sequence even under low-intensity, milliwatt-power incoherent irradiation, enabling operation at solar-level excitation.^2,3^ Provided the singlet generation yield is substantial, these characteristics render TTA-UC attractive for applications that benefit from harnessing low-energy photons, including but not limited to photovoltaics, targeted drug delivery, *in vivo* bioimaging, and 3D printing.^1,4–8^

However, now increasingly recognized, a central constraint on UC efficiency lies in the limited yield of singlet state formation *via* TTA.^9,10^ This limitation, often quantified by the spin-statistical factor (*f*), arises from the probabilistic nature of triplet–triplet encounters.^2,10–15^ The factors that ultimately dictate *f* remain actively debated, but are expected to depend on the strength of exchange coupling, relative molecular orientation, and spin-mixing pathways involving additional triplet channels.^11^

For strongly exchange-coupled triplet pairs, spin statistics dictate a singlet:triplet:quintet state ratio of 1 : 3 : 5, inherently capping *f* to 11.1%.^11,16^ Meanwhile, according to the weak exchange coupling model, spin state mixing can elevate the theoretical limit of singlet state yield up to 66%.^11^ Indeed, *f* values significantly surpassing the 11% threshold have been demonstrated experimentally^9,17–20^ with compelling evidence found in UV-emitting annihilators such as TIPS-naphthalene or PPO, which achieve remarkable TTA singlet yields exceeding 50%.^9^

Despite the considerable demand across diverse scientific and industrial applications, achieving efficient UC from the far-red/NIR to the visible spectral range remains a significant challenge.^10^ The experimentally obtained *f* for prevalent annihilators in this range, including rubrenes and diketopyrrolopyrroles, generally struggles below the 20% mark.^15,21,22^ This underscores the urgent need for innovative approaches to enhance singlet yield formation.

Notably, several experimental studies have attempted to find potential links between *f* and diverse annihilator properties. For instance, *f* appeared to be influenced by the energetic landscape and steric environment of the annihilator species.^23^ In particular, the improvement of *f* was achieved through deliberate triplet-state engineering, which ensured a substantial energy separation between 2T_1_ and higher triplet states (T_*n*_), thereby minimizing energy loss.^11,12,24^ Conversely, the incorporation of *t-*butyl side moieties in popular annihilators like rubrene and diketopyrrolopyrroles has been found to reduce the *f* factor.^21,23^ Beyond these effects, molecular geometry was also found to influence *f*, as its magnitude depends on the spatial alignment/orientation of annihilator molecules during the TTA. For example, geometric considerations could be applied to rationalize the unexpectedly high *f* values in tetracene crystals (*f* = 66%)^25^ or rigid tetracene homodimers (*f* = 40%),^26,27^ where predefined molecular alignment likely enhances TTA dynamics.^11^ Yet, despite these recent efforts to manipulate this critical parameter, accurately predicting and controlling it for new annihilators remains challenging.

In this study, we investigate the potential to tune an annihilator's spin-statistical factor *via* controlled aggregation. To this end, yellow-emitting anthradithiophene (ADT) functionalized with triethylsilyl (TES) groups was specifically chosen as the annihilator due to its triplet energy level in the near-IR range (Fig. 1b and c). Triplet sensitization of TES-ADT was accomplished using palladium phthalocyanine (PdPc) sensitizer with its triplet level positioned just above that of the annihilator, thereby ensuring efficient downward triplet energy flow. We demonstrate that a stepwise increase in annihilator concentration in toluene, triggering a shift from monomeric to aggregated states, results in a 3-fold enhancement of the singlet yield (*f*) in the TTA-UC process. This corresponds to an increase in the *f* value of TES-ADT from *ca.* 20% to 60%. Consistent with this observation, experimental data from neat solid TES-ADT films also show a similarly improved *f*. Although aggregation of the annihilator causes emission quenching and compromises overall UC efficiency, this challenge can be tackled by designing quenching-resistant molecular architectures through the incorporation of steric moieties,^15,29–31^ or by adopting alternative strategies such as the singlet sink approach.^30,32–36^

## Results and discussion

To understand how the annihilator concentration influences the UC process, we thoroughly explored the photophysical properties of TES-ADT:PdPc solutions. This involved a systematic increase in the annihilator concentration from 0.5 mM to 80 mM, while maintaining a constant PdPc concentration of 15 µM. This low sensitizer concentration has previously been shown to suppress energy back-transfer from the annihilator to the sensitizer and maximize UC performance in rubrene:PdPc solutions.^21^

The determination of *f* for each concentration relied on a comprehensive photophysical analysis, based on measured quantum yields of UC emission (*ϕ*_UC_), ISC (*ϕ*_ISC_), fluorescence (*ϕ*_FL_), TET (*ϕ*_TET_), and TTA (*ϕ*_TTA_), following the relation:1
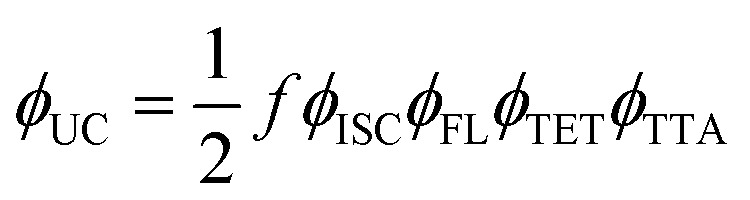


Based on this expression, the theoretical maximum for *ϕ*_UC_ is 50% when all other yields approach 100%. Given that *ϕ*_ISC_ of palladium-containing phthalocyanine derivatives is close to unity,^21,37^ only four independent parameters (*ϕ*_UC_, *ϕ*_FL_, *ϕ*_TET_ and *ϕ*_TTA_) remain to be evaluated for each annihilator concentration.

**Fig. 2:**
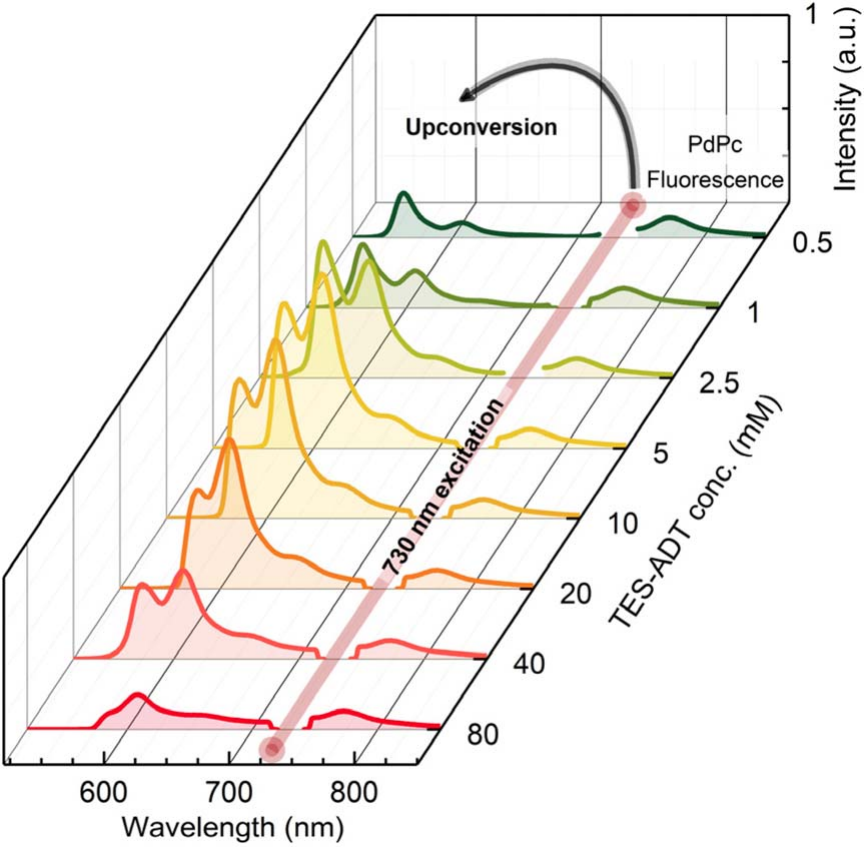
UC emission spectra of TES-ADT:PdPc in toluene as a function of annihilator concentration (0.5–80 mM) under 730 nm CW excitation. The sensitizer concentration was fixed at 15 µM. All spectra were normalized to PdPc fluorescence, assuming similar FL emission intensity, for comparison of UC efficiencies. A 730 nm notch filter was used in front of the detector to block the laser stray light.

The evolution of the UC emission spectrum with increasing TES-ADT concentration is illustrated in Fig. 2. It evidences the residual FL signal from PdPc at 760 nm alongside a prominent UC emission band peaking at 560 nm.

The intensity of the higher-energy vibronic shoulder of the UC band notably decreases with increasing annihilator concentration due to self-absorption effects. Additionally, the non-monotonic behavior of this band, marked by an initial increase in intensity followed by a decline, unveils intricate photophysics to be discussed in detail below.

**Fig. 3:**
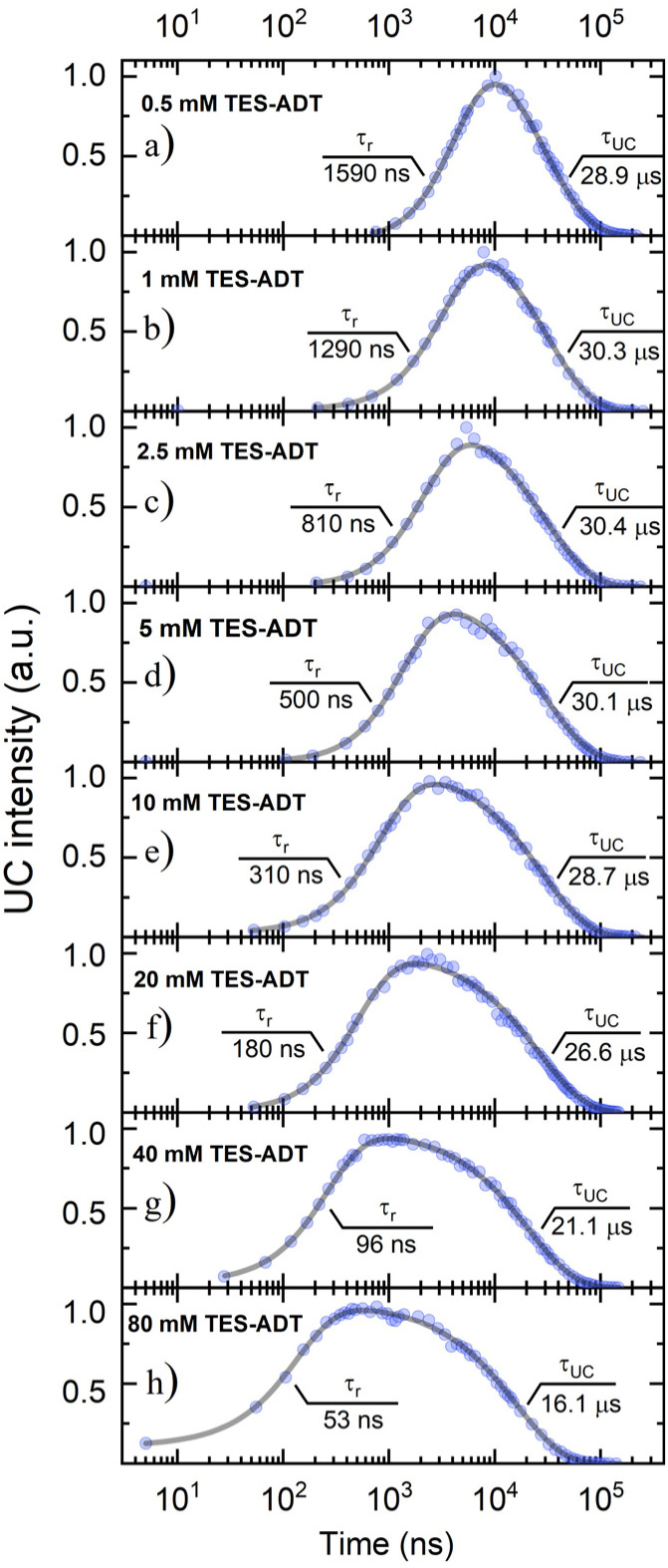
UC transients of TES-ADT:PdPc in toluene at various annihilator concentrations (as indicated) under 730 nm pulsed excitation. The sensitizer concentration was fixed at 15 µM. Solid lines represent fits based on eqn (S1), with the extracted UC signal rise (*τ*_r_) and decay (*τ*_UC_) time constants indicated.

*ϕ*_TET_ was evaluated from the UC emission transients (Fig. 3), assuming that the primary decay pathway for sensitizer triplets is their transfer to the annihilator, which manifests as a rise in the UC signal. This implies that the rise-time of UC intensity (*τ*_*r*_) corresponds to the decay time of the sensitizer triplets (*τ*_PdPc_), as described by the relation 
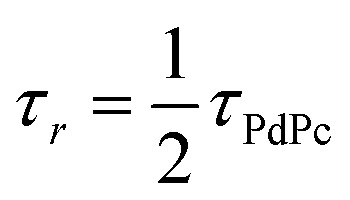
, derived from eqn (S1). The values of *τ*_PdPc_ at each TES-ADT concentration (see Fig. S1a) were extracted and used to calculate *ϕ*_TET_ as defined by:2
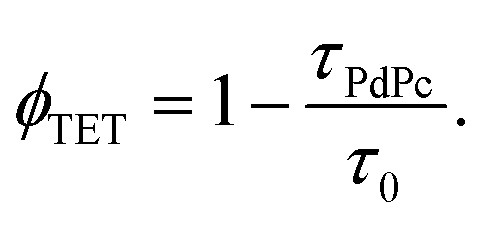
Here, *τ*_0_ denotes the intrinsic triplet lifetime of the sensitizer in the absence of the annihilator, determined from a linear fit of the inverse PdPc lifetime as a function of TES-ADT concentration (Fig. S1b). The obtained *τ*_0_ value of 3.78 µs for PdPc is in excellent agreement with previously reported results.^21^ The expected trend of *τ*_*r*_ shortening with increasing TES-ADT molar concentration, corresponding to reduced intermolecular spacing, was observed, indicating improved TET efficiency from the sensitizer to annihilator (Fig. 5b). Notably, *ϕ*_TET_ only became significantly high (>80%) above 10 mM of TES-ADT. Furthermore, UC transient data at low annihilator concentrations revealed a consistent UC decay time (*τ*_UC_) of ∼30 µs, which dropped abruptly above 10 mM concentration (Fig. 3). Given that 
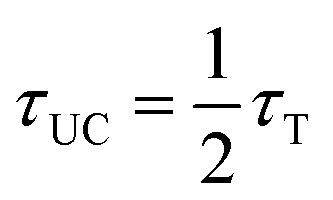
 (as derived from eqn (S1)), this drop reflects a considerable shortening in the TES-ADT triplet lifetime (*τ*_T_), hinting at potential annihilator aggregation.

*ϕ*_FL_ and *ϕ*_UC_ for TES-ADT:PdPc solutions were determined under steady-state excitation conditions using an integrating sphere and a comparative method, respectively, as detailed in Fig. S2–S4. The quantum yields were corrected for the spectral distortions caused by PdPc and TES-ADT self-absorption. Unlike the increasing trend observed for TET efficiency, *ϕ*_FL_ showed gradual quenching with higher TES-ADT concentrations, attributed to enhanced non-radiative decay due to annihilator aggregation (Fig. 5b). Despite the monotonic decrease of FL yield, *ϕ*_UC_ followed a bell-shaped profile, reaching a maximum value of 6.8% at 5 mM TES-ADT concentration (Fig. 5a). This annihilator concentration highlights an optimal point where the trade-off between improved TET efficiency and FL quenching is balanced.

**Fig. 4:**
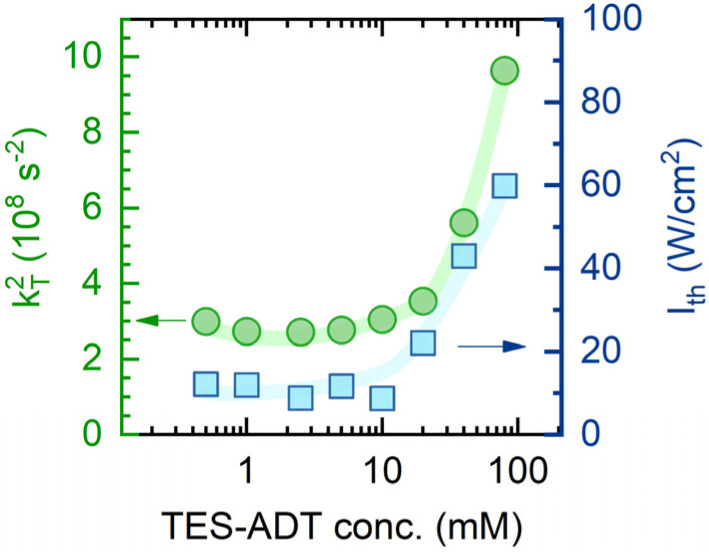
Dependence of the square of TES-ADT triplet decay rate (*k*_T_^2^, circles) and UC threshold (*I*_th_, squares) on annihilator molar concentration. *k*_T_ was determined from the UC decay transients presented in Fig. 3. Lines are guides to the eye.

Lastly, to account for *ϕ*_TTA_ in eqn (1), UC intensity as a function of excitation power density (*I*_ex_) was measured at various annihilator concentrations and modelled using the procedure described by Murakami *et al.*^2^ (Fig. S5). The excellent fit of the experimental data enabled determination of the saturated UC quantum yield (*ϕ*^∞^_UC_) and UC threshold (*I*_th_). The sudden increase of *I*_th_ observed at annihilator concentrations above 10 mM (squares in Fig. 4) was primarily attributed to the accelerated triplet decay rate *k*_T_ (=1/*τ*_T_) of TES-ADT (circles in Fig. 4), as described by eqn (3):3
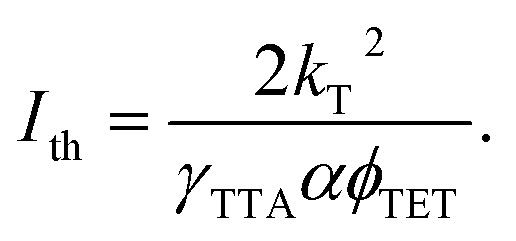


**Fig. 5:**
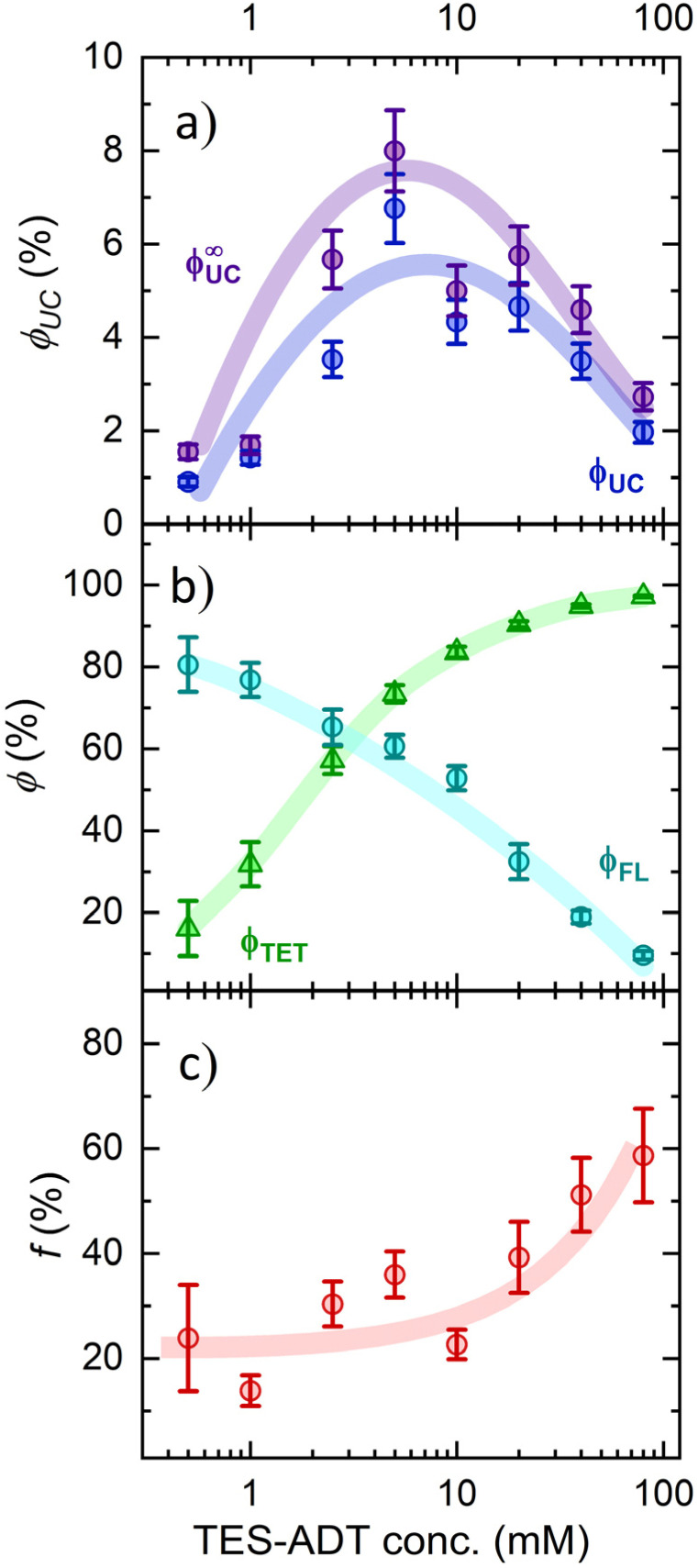
Dependence of key photophysical parameters of TES-ADT:PdPc solutions on TES-ADT concentration: (a) UC quantum yields (*ϕ*_UC_ and *ϕ*^∞^_UC_), (b) FL and TET quantum yields (*ϕ*_FL_ and *ϕ*_TET_, respectively), (c) spin-statistical factor. Lines are guides to the eye.

The obtained *ϕ*^∞^_UC_ represents the maximum attainable *ϕ*_UC_ for the corresponding annihilator concentration, achieved in the limit where *I*_ex_ approaches infinity (Fig. 5a). Under such excitation conditions, TTA dominates over spontaneous triplet relaxation, leading to *ϕ*_TTA_ = 1. Thus, by substituting UC yield with *ϕ*^∞^_UC_ values in eqn (1) and solving for *f*, the equation can be simplified to the following form:4
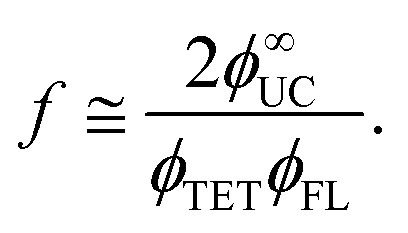


The spin-statistical factor, determined from eqn (4), was found to increase from *ca.* 20% to 60% with rising TES-ADT concentration (see Fig. 5c and Table 1). The uncertainty analysis provided in the SI confirms that this threefold increase in *f* is robust within the experimental error. Interestingly, this trend became noticeably steeper at concentrations exceeding 10 mM, a behaviour we previously linked to potential annihilator aggregation.

**Table 1:** Main UC properties of TES-ADT:PdPc film and UC solutions with varying TES-ADT concentration

TES-ADT conc. (mM)	*ϕ*_UC_ (%)	*I*_th_ (W cm^−2^)	*ϕ* ^∞^_UC_ (%)	*ϕ*_FL_ (%)	*ϕ*_TET_ (%)	*f* (%)
0.5	0.9	12.2	1.5	80.5	16.1	23.1
1	1.4	11.9	1.7	76.8	31.8	13.9
2.5	3.5	8.8	5.7	65.3	57.2	30.5
5	6.8	11.7	8.0	60.6	73.4	36.0
10	4.3	8.7	5.0	52.8	83.6	22.7
20	4.7	22.1	5.8	32.4	90.4	39.6
40	3.5	43.0	4.6	18.9	94.9	51.3
80	2.0	59.9	2.7	9.6	97.2	57.9
UC film	0.25	8.9	0.4	1.6	94.4	53.0

**Fig. 6:**
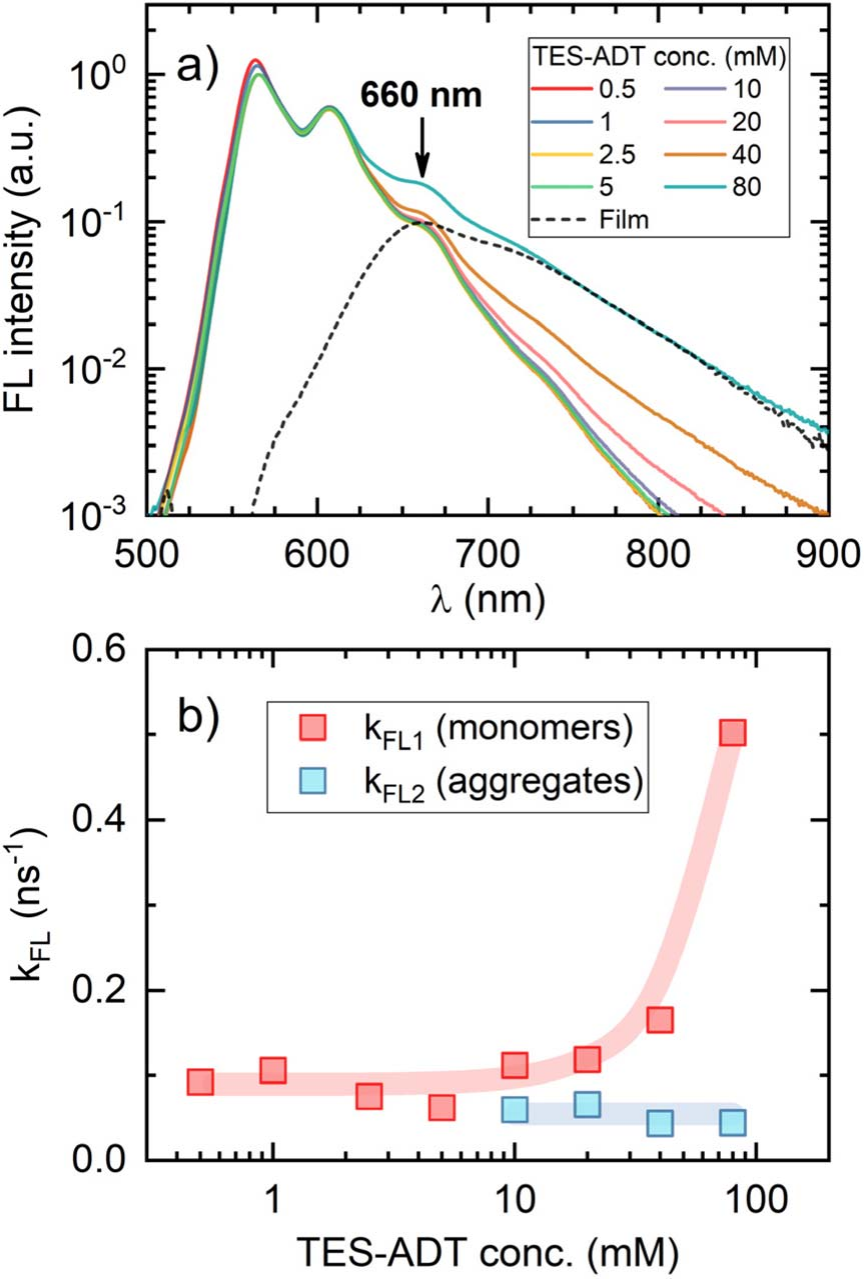
(a) FL spectra of TES-ADT in toluene at different concentrations (indicated). The dashed line shows the spectrum of a neat TES-ADT film (with the peak marked by an arrow), normalized to the long-wavelength slope of the 80 mM solution spectrum. (b) FL decay rates of monomolecular and aggregate species of TES-ADT in toluene as a function of concentration, following excitation at 510 nm and detection at 660 nm. Beyond 10 mM concentration, FL decay becomes bi-exponential with respective rate constants *k*_FL1_≡*τ*_FL1_^−1^ and *k*_FL2_≡*τ*_FL2_^−1^. Notable increase in *k*_FL1_ above the 10 mM TES-ADT is attributed to enhanced energy transfer to low-lying aggregate states.

To clarify the increasing trend in *f* value as a function of TES-ADT concentration, we prepared a dedicated series of toluene solutions of TES-ADT that were sensitizer-free. FL spectral dynamics of these solutions with increasing annihilator concentration clearly demonstrated the emergence of a new band on the long-wavelength slope of the monomolecular band (Fig. 6a). At the highest TES-ADT concentrations, the long-wavelength slope of this new band matched that of the neat film PL spectrum (peaking at 660 nm), further supporting its aggregate-related origin. Close inspection of FL transients of the same series of solutions revealed bi-exponential FL decay above 10 mM TES-ADT (Fig. S6 and Table S1), indicating two distinct FL decay rates, *k*_FL1_ and *k*_FL2_, likely associated with TES-ADT monomers and aggregates (Fig. 6b), respectively. This observation, combined with the drop in *ϕ*_FL_ (Table 1) and the accelerated triplet decay rate *k*_T_ at concentrations exceeding 10 mM (Fig. 4), provides additional evidence for annihilator aggregation and the resulting quenching. It is therefore apparent that the increase in the spin-statistical factor with rising annihilator concentration is directly linked to this aggregation, unveiling aggregation-enhanced singlet generation *via* TTA in TES-ADT molecules.

These claims were further substantiated by determining the *f* value in a neat TES-ADT film doped with a low concentration (0.1 wt%) of PdPc sensitizer. This concentration was specifically chosen to mitigate sensitizer aggregation and back-FRET effects.^22,30^ Due to a poor *ϕ*_FL_ value of 1.6% caused by aggregation-quenching, the film demonstrated a moderately low *ϕ*_UC_ of 0.25% and the saturated UC quantum yield of *ϕ*^∞^_UC_ = 0.4% (Fig. S7 and S8). While these figures may seem modest, they are comparable to values previously reported by Kamada *et al*.^36^ The *f* value for this film was estimated at (53.0 ± 10.6)%, which closely matches the values observed in high annihilator concentration UC solutions (Fig. 5c), thereby confirming that the spin-statistical factor is indeed significant in the solid-state. Note that the film *ϕ*_UC_, and thus inferred *f* value, may be slightly inflated by annihilator triplet recycling mediated by back-FRET from the annihilator to the sensitizer. To address this, we developed a simple steady-state model that explicitly includes back-FRET–driven triplet recycling (see SI). Using the experimentally estimated back-FRET efficiency, we obtained an intrinsic spin-statistical factor of *f*_*i*_ = 49.0%, corresponding to an effective inflation of 4 percentage points relative to the *f* value extracted without recycling. This correction remains within our experimental uncertainty and does not affect the conclusions.

To address the origin of this *f* enhancement, we considered the possible redistribution of energy levels (or a change in the energy landscape) of TES-ADT upon aggregation. Consequently, we performed density functional theory (DFT) calculations to determine the relative positions of the levels for both monomer and dimer TES-ADT. For the monomer, ground-state geometry optimization and time-dependent DFT calculations were performed utilizing a B3LYP functional and a 6-311G(d) basis set. The ground-state geometry of the dimer was obtained from experimental XRD data of a TES-ADT crystal. Comprehensive TES-ADT crystallographic data, including molecular geometry and packing arrangements, are presented in the SI (Table S2 and Fig. S9).

Based on these data, dimerization was found to cause a redshift and splitting of energy levels (Fig. S10). Additionally, a few new dimer-related states of singlet and triplet character emerged within the 1.94–1.98 eV range. The *ca.* 460 meV energy difference between the S_2_ state and the newly identified dimer state S_1_ is noteworthy, as it roughly corresponds to the difference between the emission maximum of the dilute TES-ADT solution (565 nm) and the neat film (660 nm), as shown in Fig. 6a. This correlation supports the relative energy level alignment predicted by DFT.

In the case of the TES-ADT monomer, T_2_ is largely unreachable through TTA at room temperature due to an energy difference of 90 meV (T_2_ – 2T_1_). Conversely, in the dimer, higher-energy triplet states, up to T_6_, are fully accessible during the annihilation, suggesting their possible involvement in the UC process.^38^ To assess the role of these T_*n*_ states in spin-conversion, we calculated spin–orbit coupling (SOC) constant between S_1_, S_2_ and the T_1_ – T_6_ states. The calculations revealed extremely weak coupling with negligible SOC (<0.05 cm^−1^) for most T_1−6_ → S_1,2_ transitions, except for the energetically viable T_6_ → S_1_ and T_6_ → S_2_ transitions, yielding notable SOC values of 0.44 cm^−1^ and 0.16 cm^−1^, respectively (Table S3). We propose that these moderate SOC transitions, absent in the monomeric form, facilitate the conversion process, resulting in a 3-fold enhancement of the singlet yield compared to TES-ADT monomers (Fig. 5c). Notably, such high-level reverse intersystem crossing (HL-RISC) has also been proposed as an additional spin-conversion pathway in a few previous reports on both OLEDs and TTA-UC systems.^11,19,38,39^ In TTA emitters, experiments on fluorinated phenazine/acridine annihilators with accessible high-energy triplet states^38^ and on DPA derivatives with tailored molecular connectivity and rigidity^19^ showed that tuning the energies and population of these upper triplets can open additional HL-RISC channels and increase, or even surpass, the nominal spin-statistical limit. A complementary spin-statistics model^11^ explicitly incorporates higher triplet levels, whose energies and mixing with the singlet manifold depend on intermolecular orientation and exchange coupling, thereby rationalizing the enhanced singlet generation in acene-based systems. Our DFT results, which place higher-lying triplet states (*e.g.*, T_6_) close in energy to S_1_/S_2_, naturally fit within this HL-RISC framework and suggest that the enhanced singlet generation observed for aggregated TES-ADT arises from an increased contribution of such upper triplet states during the TTA encounter. This enhancement in singlet generation may also extend to other annihilators in the aggregated form, presenting an intriguing topic for future research.

## Conclusions

This study addresses the low yield of singlet state formation during TTA, a primary limitation to efficient photon upconversion, particularly in the desirable far-red/NIR spectral range. We tackled this by tuning the spin-statistical factor (*f*) of TES-ADT annihilator solutions through concentration-induced aggregation.

Our experiments demonstrate a 3-fold enhancement in the singlet yield, boosting the *f* value of TES-ADT from *ca.* 20% to 60% with increasing annihilator concentration. This is shown to correlate with the onset of annihilator aggregation, as evidenced by the appearance of new emission bands, bi-exponential fluorescence transients, and accelerated triplet decay rates. Furthermore, the similarly high *f* value observed in a neat TES-ADT film supports the role of aggregation in enhancing singlet yield. DFT calculations suggest that TES-ADT dimerization renders access to higher-energy triplet states (up to T_6_) *via* TTA, with moderate spin–orbit coupling to the singlet manifold likely promoting spin conversion in the dimers. This work, thus, showcases a new approach to overcoming the spin-statistical limitations of TTA-UC by leveraging aggregation-enhanced singlet generation through molecular packing and the resulting modulation of the annihilator energy landscape.

## Experimental

### Materials

The synthesis of palladium phthalocyanine (PdPc) was published elsewhere.^21^ TES-ADT was purchased from Sigma-Aldrich.

### DFT calculations

The molecular geometry and singlet/triplet state energies of the TES-ADT annihilator were modelled using the ORCA 5.0 software package.^40^ Geometry optimizations were carried out in vacuum at the B3LYP/6-311G(d) level of DFT. Time-dependent DFT was then applied to the optimized structure to determine the singlet and triplet excited-state energies.

### Sample preparation

UC solutions and the film were prepared in a nitrogen-filled glovebox, maintaining water and oxygen levels below 0.1 ppm. Toluene solutions of TES-ADT:PdPc were obtained by mixing the annihilator and sensitizer at appropriate ratios to yield final TES-ADT concentrations of 0.5, 1, 2.5, 5, 10, 20, 40, and 80 mM, with a fixed PdPc concentration of 15 µM. Screw-cap quartz cuvettes (1 × 10 mm) containing UC solutions were carefully sealed by wrapping the cap–neck interface with 5–6 layers of Parafilm prior to removal from the glovebox and subsequent photophysical measurements. UC film was fabricated by drop-casting a concentrated 10 mg mL^−1^ TES-ADT stock solution in toluene containing 0.1 wt% PdPc, followed by encapsulation between two thin glass plates using epoxy resin.

### Optical techniques

Absorption spectra of dilute annihilator and sensitizer solutions were recorded using an FLS980 spectrometer (*Edinburgh Instruments*). All steady-state emission spectra were measured with a back-thinned CCD spectrometer PMA-12 (*Hamamatsu*) using continuous-wave 510 nm and 730 nm lasers (*PicoQuan*t) as excitation sources. FL quantum yields were determined by utilizing an integrating sphere (*Sphere Optics*) coupled to the CCD spectrometer *via* an optical fiber.^41^ UC quantum yields were evaluated using a comparative method (see SI). FL transients were measured using a time-correlated single-photon counting system PicoHarp 300 (*PicoQuant*) with the same lasers operated in pulsed mode. UC transients were recorded with a time-gated iCCD camera iStar DH340T (*Andor*) mounted on a spectrograph SR-303i (*Shamrock*) and excited using a YAG:Nd^3+^ laser NT242 (*Ekspla*) equipped with an optical parametric oscillator (730 nm excitation wavelength, 1 kHz repetition rate, 5 ns pulse duration) as the excitation source.

### Crystallographic analysis

Single crystals for XRD analysis were grown by the slow evaporation of toluene solution. Suitable crystals were selected and analyzed on an XtaLab Synergy diffractometer (Rigaku), equipped with a HyPix-6000HE hybrid photon counting detector and a PhotonJet microfocus X-ray source (CuKα, *λ* = 1.54184 Å). Data was collected and processed using the CrysAlisPro software. The structures were solved by Intrinsic Phasing with the ShelXT^42^ program and refined with the ShelXL^43^ package, using least-squares minimization and employing the Olex2 graphical interface.^44^ The structure file of the TES-ADT crystal was deposited with the Cambridge Crystallographic Data Centre and is available free of charge (CCDC 2471956).

## Author contributions

KK, JL, and ER conceptualized the idea of this work. JL performed experimental measurements, DFT calculations, and prepared the initial draft of the manuscript. AJ and EO synthesized the materials. GK carried out XRD measurements and crystallographic analysis. KK and ER mainly developed and revised the manuscript. All authors have given approval to the final version of the manuscript.

## Conflicts of interest

There are no conflicts to declare.

## Supplementary Material

SC-OLF-D5SC07013A-s001

SC-OLF-D5SC07013A-s002

## Data Availability

CCDC 2471956 contains the supplementary crystallographic data for this paper.^45^ The data supporting this article have been included as part of the supplementary information (SI). Supplementary information: experimental details and additional UC/FL spectra and transient analyses (TET yields, reabsorption-corrected quantum yields, power-dependence/threshold fits), solid-film spin-statistical factor evaluation, TES-ADT crystallographic data, TD-DFT results, and error analysis. See DOI: https://doi.org/10.1039/d5sc07013a.
