# Supplementary material for: Aggregation favors singlet formation in TES-ADT triplet annihilator for photon upconversion
Source: Chem Sci. 2026 Jan 28;17(12):6230–7. doi: 10.1039/d5sc07013a (PMC12871387; doi:10.1039/d5sc07013a)
Supplement: SC-017-D5SC07013A-s001 [file SC-017-D5SC07013A-s001.pdf]

Supplementary Information (SI) for Chemical Science.
This journal is © The Royal Society of Chemistry 2026

Supplementary information

Aggregation Favors Singlet Formation in TES-ADT Triplet Annihilator for Photon Upconversion

Justas Lekavičius, [a] Edvinas Radiunas, [a] Gediminas Kreiza, [a] Augustina Jozeliūnaitė, [b] Edvinas Orentas [b] and Karolis Kazlauskas *[a]

[a] Institute of Photonics and Nanotechnology, Vilnius University, Saulėtekio av. 3, LT-10257 Vilnius, Lithuania.
[b] Institute of Chemistry, Faculty of Chemistry and Geosciences, Vilnius University, Naugarduko 24, LT-03225 Vilnius, Lithuania

*e-mail: karolis.kazlauskas@ff.vu.lt

Determination of TET quantum yields from UC transients

UC transients measured at low excitation power density were approximated by the following function:[1]

$$I_{UC}(t) \propto [T_A]^2 = \left(A \cdot exp\left(-\frac{t}{\tau_{PdPc}}\right) - B \cdot exp\left(-\frac{t}{\tau_T}\right)\right)^2, \qquad (S1)$$

where τ_{PdPc} and τ_T represent sensitizer and annihilator triplet decay times, respectively. From this function, UC rise (τ_r) and decay (τ_{UC}) times can be calculated as $\tau_r = \frac{1}{2}\tau_{PdPc}$ and $\tau_{UC} = \frac{1}{2}\tau_T$.

Triplet energy transfer yield (ϕ_{TET}) was determined from the rise transients of the UC signal using the quenched sensitizer lifetime (τ_{PdPc}) and the natural triplet lifetime of the sensitizer (τ_0) via the following equation:

$$\phi_{TET} = 1 - \frac{\tau_{PdPc}}{\tau_0}. \qquad (S2)$$

τ_0 was extrapolated from a linear fit of the sensitizer triplet decay rate (τ_{PdPc}^{-1}) plotted against the annihilator concentration.

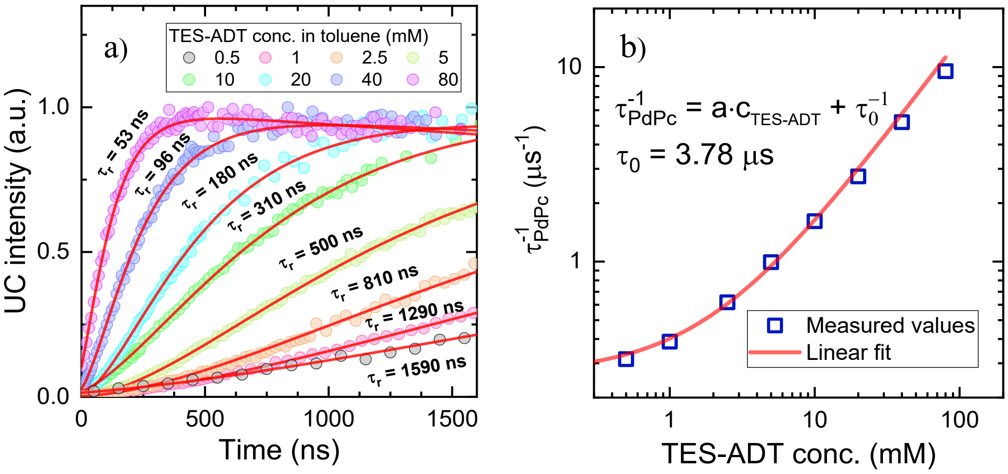

Fig. S1 a) UC emission rise profiles of TES-ADT:PdPc solutions with a constant sensitizer concentration (15 µM). $\tau_r (= 0.5\tau_{PdPc})$ is the UC signal rise time. b) Dependence of the sensitizer decay rate (τ_{PdPc}^{-1}) on TES-ADT concentration. A linear fit in the region of 0.5 to 10 mM of TES-ADT was used to determine the intrinsic triplet lifetime (τ_0) of the sensitizer.

FL quantum yields of TES-ADT:PdPc solutions (corrected for reabsorption)

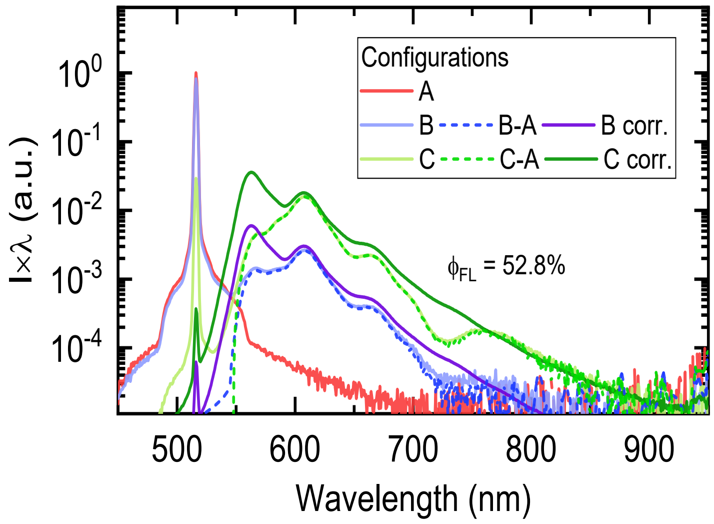

Fig. S2 Representative data set for FL quantum yield evaluation with an integrating sphere, featuring the UC solution containing 10 mM TES-ADT and 15 µM PdPc. 510 nm CW laser was employed for excitation. In A configuration, the integrating sphere contains only a toluene-filled cuvette as a reference. In B configuration, the sample is excited only by the scattered laser light, whereas in C configuration, the excitation beam passes directly through the

sample. B-A and C-A spectra were obtained by subtracting the reference spectrum (A), serving as a background, from the B and C spectra. B corr. And C corr. Refer to TES-ADT emission spectra measured outside the sphere, which were used to correct for spectral distortions caused by PdPc absorption (around 650 and 730 nm) and TES-ADT self-absorption (around 550 nm) within the integrating sphere. This correction procedure was applied consistently across all TES-ADT concentrations to obtain corrected ϕ_{FL}.

UC quantum yields of TES-ADT:PdPc solutions (corrected for reabsorption)

UC quantum yields of the samples were evaluated using a comparative method relative to PdPc fluorescence. 15 µM PdPc solution in toluene, matching the PdPc concentration in the UC solutions, served as the reference with a known FL quantum yield of $\phi_{PdPc} = 0.36\%$. Given that the absorption coefficients of the reference and UC solutions at 730 nm are equal, ϕ_{UC} was calculated using the following formula:

$$\phi_{UC} = \phi_{PdPc} \frac{I_{UC}}{I_{PdPc}}, \qquad (S3)$$

where I_{UC} and I_{PdPc} are spectrally integrated UC and FL_{PdPc} intensities of the UC solution and reference, respectively, each multiplied by the emission wavelength. ϕ_{UC} values for each solution were measured at different excitation power densities, as shown in Fig. S5.

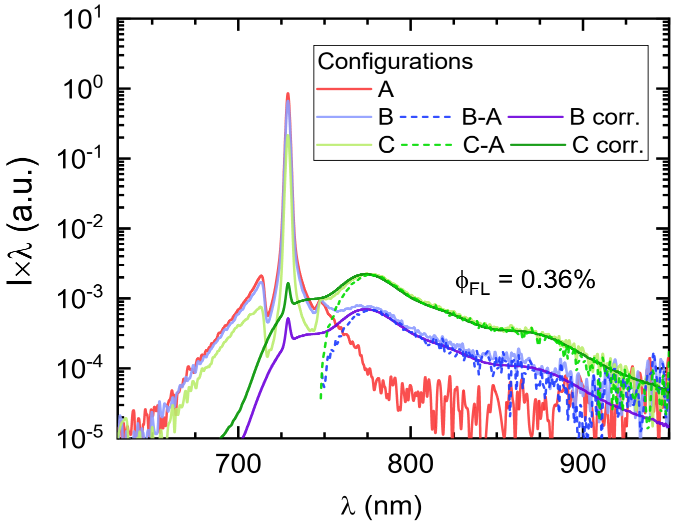

Fig. S3 Set of spectra used for FL quantum yield evaluation of 15 µM PdPc solution in toluene (a reference solution) with an integrating sphere. A 730 nm notch filter was placed in front of the detector to reduce the intensity of the laser stray light by a factor of 20.64 (measured separately).

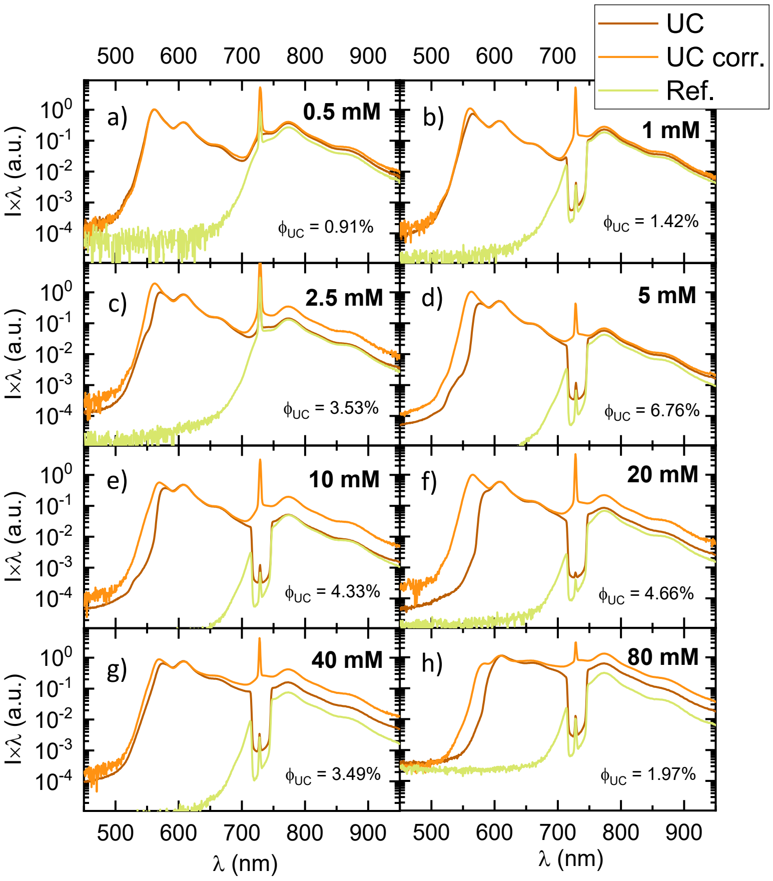

Fig. S4 (a-h) UC quantum yield estimations for UC solutions (TES-ADT:PdPc) at different annihilator concentrations (indicated), measured by a relative method with 15 μM PdPc toluene solution as the reference ($\phi_{FL} = 0.36\%$). Emission spectra of the UC solutions (brown lines) and the reference solution (green lines) were recorded under identical experimental conditions by exciting the center of a 1-mm-thick quartz cuvette. Additional UC spectra (yellow lines) were obtained by exciting the edge of the same cuvette and were used to correct for TES-ADT self-absorption.

Determination of saturated UC quantum yield and threshold

The saturated UC quantum yield (ϕ_{UC}^{∞}), defined as the yield at infinitely high excitation power density, and the excitation threshold (I_{th}) were determined from measurements of UC signal intensity as a function of excitation power density (I_{ex}). The data were converted to ϕ_{UC} versus I_{ex}, considering that

$$\phi_{UC} \propto \frac{I_{UC}}{I_{ex}} \quad (S4)$$

and rescaled using known ϕ_{UC} values (see the previous section). The relevant parameters were extracted by fitting the experimental data to the function proposed by *Murakami et al.*:[2]

$$\phi_{UC} = \phi_{UC}^{\infty}\left(1 + \frac{1-\sqrt{1+4I_{ex}/I_{th}}}{2I_{ex}/I_{th}}\right). \qquad (S5)$$

In this model, I_{th} corresponds to the excitation power density at which $\phi_{UC} = 0.382 \cdot \phi_{UC}^{\infty}$.

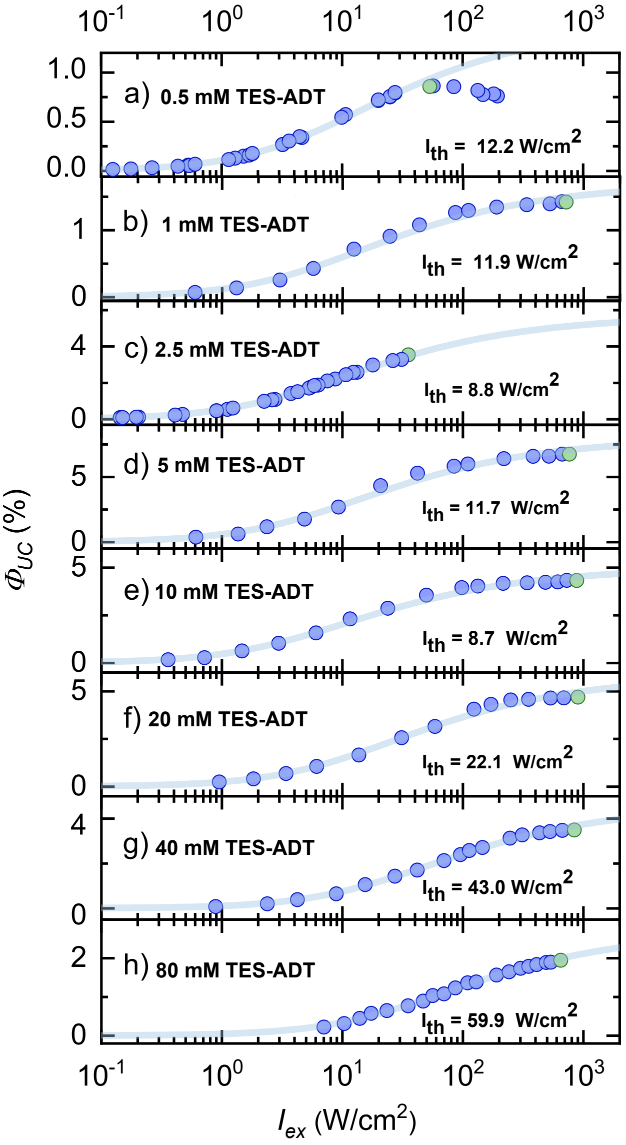

Fig. S5 (a-h) UC quantum yield as a function of excitation power density I_{ex} for UC solutions with varying TES-ADT concentration. ϕ_{UC} values measured using a relative method are marked in green.

FL transients of TES-ADT in toluene

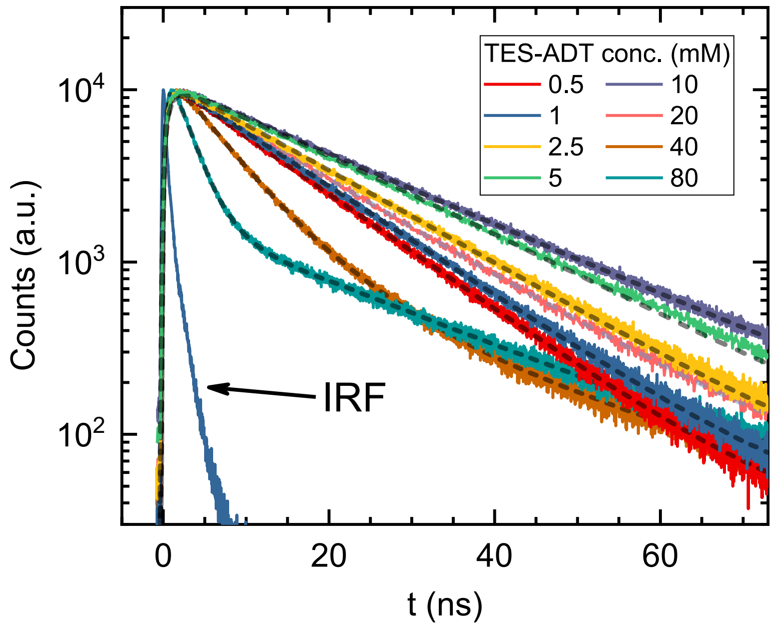

Fig. S6 FL transients of sensitizer-free TES-ADT in toluene at various concentrations (indicated), excited at 510 nm and detected at 660 nm. IRF, instrument response function. Dashed lines represent single- or multi-exponential fits of the measured transients. The extracted decay lifetimes (τ_1 and τ_2), along with their fractional contributions (c_1 and c_2), are summarized in Table S1.

Table S1. FL decay lifetimes τ_i and fractional contributions of each decay component c_i to the overall decay of TES-ADT in toluene.

TES-ADT conc. (mM)	τ_1, ns	c_1, %	τ_2, ns	c_2, %
0.5	10.8	100	–	–
1	9.4	100	–	–
2.5	13.2	100	–	–
5	15.9	100	–	–
10	8.9	55.20	16.6	44.80
20	8.4	20.36	15.1	79.64
40	6.0	72.52	22.8	27.48
80	2.0	45.35	22.4	54.65

The slight increase in the FL decay lifetime (τ_1) of monomolecular TES-ADT species with rising concentration from 0.5 to 5 mM is attributed to enhanced re-absorption and re-emission effects. The subsequent rapid reduction of this lifetime (τ_1) at TES-ADT concentrations above 10 mM is due to energy transfer from single molecules to lower-energy aggregate states. The appearance of the second FL decay component above 10 mM TES-ADT, with a lifetime (τ_2) in the range of 15-23 ns, is specifically associated with TES-ADT aggregates.

Spin-statistical factor of TES-ADT:PdPc solid film

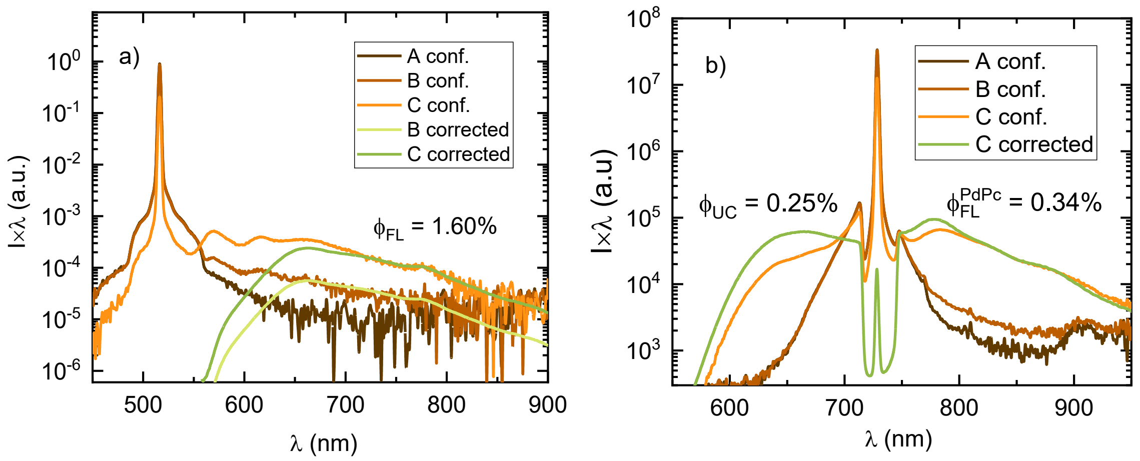

Fig. S7. Evaluation of (a) FL and (b) UC quantum yields for a neat TES-ADT film doped with 0.1 wt% PdPc. Both yields were measured using an integrating sphere. The samples were laser-excited at 510 nm for ϕ_{FL} and at 730 nm for ϕ_{UC}. 730 nm notch filter was placed in front of the detector for ϕ_{UC} measurement to reduce the laser stray light by a factor of 41 (measured separately). FL signal observed between 550 and 650 nm in the integrating sphere measurements (B and C conf.) is attributed to single-molecule emission, likely caused by indirect excitation of the sample edges. This signal was excluded from the final ϕ_{FL} calculations, with the true spectral shape taken from separate correction measurements performed outside the integrating sphere.

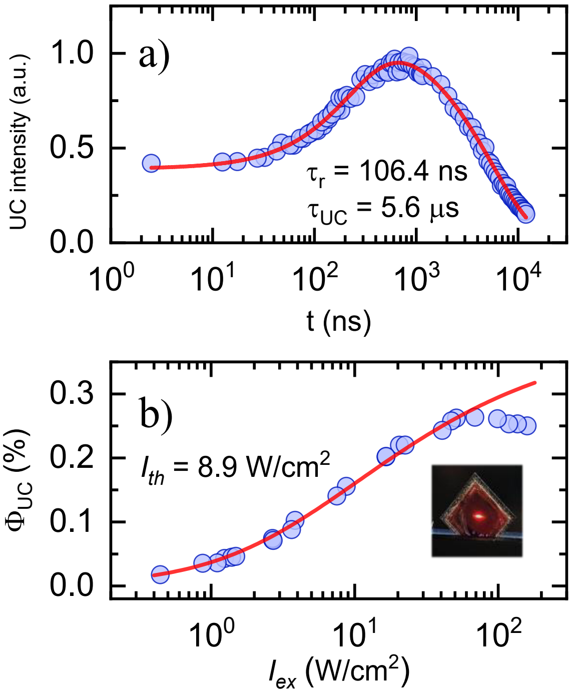

Fig. S8 a) UC transients and (b) ϕ_{UC} as a function of excitation power density (I_{ex}) for neat TES-ADT film doped with 0.1 wt% PdPc. The data was fitted up to 50 W/cm^2, as experimental values at higher power densities are affected by sample photodegradation. τ_r – signal rise time, $\tau_{UC} = \frac{\tau_T}{2}$ – UC decay time, I_{th} - excitation threshold power density.

TES-ADT crystallographic data

Table S2. Crystallographic data of TES-ADT crystal.

Formula	$C_{34} H_{38} S_2 Si_2$
$D_{calc.}$ (g·cm^{-3})	1.154
μ (mm^{-1})	2.325
Formula Weight	566.94
Colour	Red
Shape	Plate
Size (mm^3)	0.41×0.23×0.04
Temperature (K)	300.1(7)
Crystal system	triclinic

Space group	$P\bar{1}$
a (Å)	6.9092(2)
b (Å)	7.4194(2)
c (Å)	16.6957(5)
α (deg)	96.226(2)
β (deg)	91.986(2)
γ (deg)	106.077(2)
Volume (Å^3)	815.66(4)
Z	1
Z'	0.5
Wavelength (Å)	1.54184
Radiation type	Cu K_α
Θ_{min} (deg)	2.668
Θ_{max} (deg)	77.119
Measured Refl.	7857
Independent Refl.	3287
Reflections with $I > 2(I)$	2862
R_{int}	0.0247
Parameters	175
Restraints	3
Largest Peak	1.762
Deepest Hole	-0.768
GooF	1.865
wR_2 (all data)	0.3991
wR_2	0.3878
R_1 (all data)	0.1193
R_1	0.1122

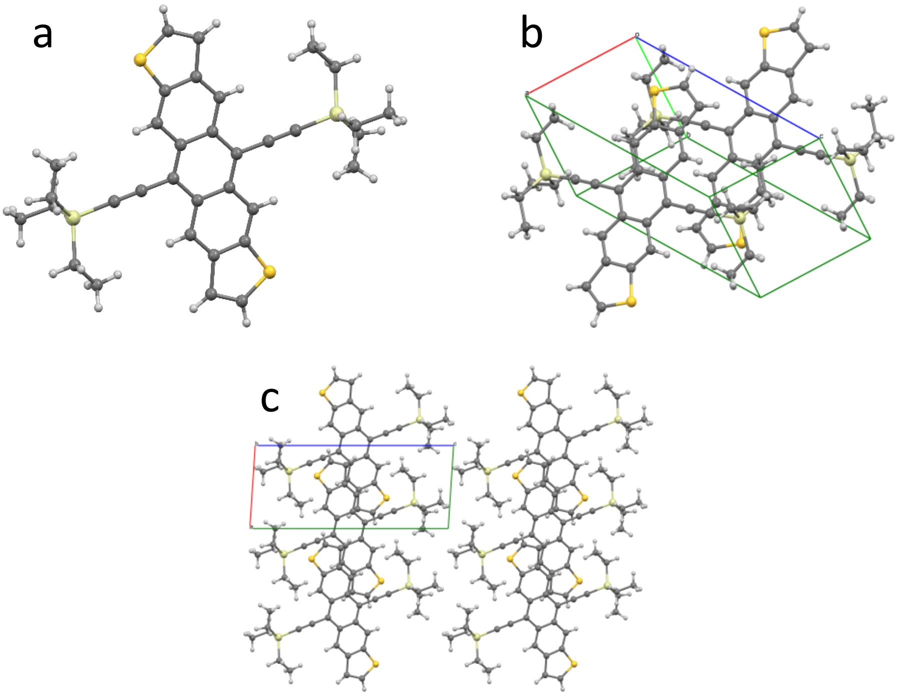

Fig. S9 Molecular geometry (a) and packing (b-c) of TES-ADT crystals.

DFT calculations

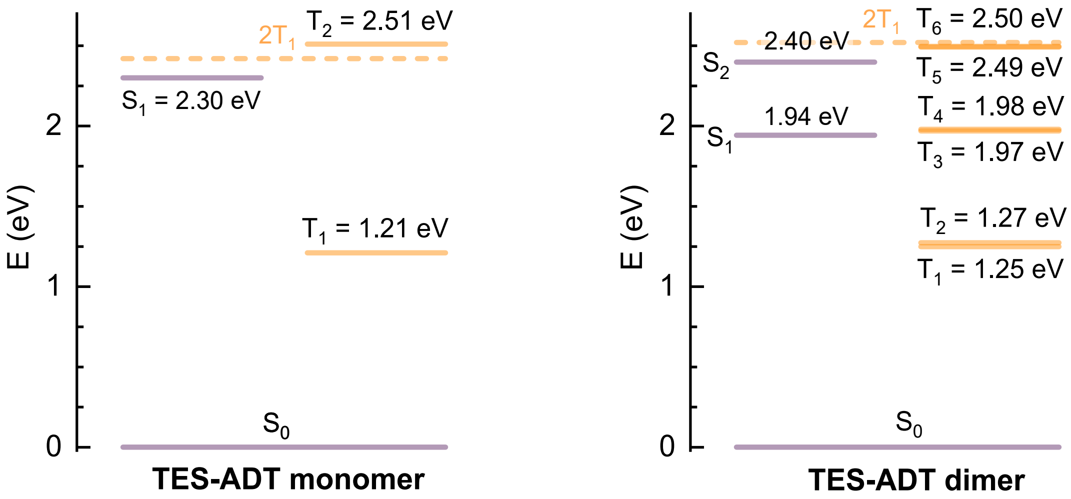

Fig. S10 Transition energies from ground to excited singlet and triplet states of TES-ADT monomer and dimer, calculated at TD-DFT level. TES-ADT monomer ground state geometry optimization and TD-DFT calculations were performed using B3LYP functional and 6-311G(d) basis set. TES-ADT dimer geometry was obtained from experimental XRD data of a TES-ADT crystal.

Table S3. Calculated SOC matrix elements between excited triplet and singlet states for TES-ADT dimer using XRD-derived geometry.

**Molecular state**		**SOC matrix elements (cm^{-1})**
T_1	S_1	0.03
**T_1**	**S_2**	**0.21**
T_2	S_1	0.00
T_2	S_2	0.00
T_3	S_1	0.00
T_3	S_2	0.00
T_4	S_1	0.04
**T_4**	**S_2**	**0.14**
T_5	S_1	0.00
T_5	S_2	0.00
**T_6**	**S_1**	**0.44**
**T_6**	**S_2**	**0.16**

According to the energy scheme in Fig. S10, the energetically viable transitions for the TES-ADT dimer are $\boldsymbol{T_6} \rightarrow \boldsymbol{S_1}$ and $\boldsymbol{T_6} \rightarrow \boldsymbol{S_2}$.

Error analysis

Triplet energy transfer yield ($\boldsymbol{\phi_{TET}}$).

Using Eq. (S2), the uncertainty was obtained by standard error propagation from the fitted PdPc lifetimes in the UC sample, τ_{PdPc}, and the intrinsic PdPc lifetime, τ_0:

$$\Delta\phi_{TET} = \sqrt{\left(\frac{\Delta\tau_{\mathrm{PdPc}}}{\tau_0}\right)^2 + \left(\frac{\tau_{PdPc}}{\tau_0^2}\Delta\tau_0\right)^2}\,. \qquad (S6)$$

The uncertainty is derived from fits of the UC transient decays for each concentration (Fig. S1a and Fig. 3), while $\Delta\tau_0$ is common to all samples and obtained from the fit in Fig. S1b ($\Delta\tau_0 =$ 299 ns).

Fluorescence quantum yield (ϕ_{FL}).

The dominant uncertainty originates from scaling the correction spectrum C_{corr} to match the spectrum measured inside the integrating sphere. Variations in B_{corr} were found to have a negligible impact. We therefore determined limiting cases (ϕ_{FL}^{low} and ϕ_{FL}^{high}) by varying the C_{corr} scaling within the range, yielding physically reasonable spectral matching, and used their spread to calculate $\Delta\phi_{FL}$. An example of spectral matching under two limiting cases is shown in Fig. S11 for 15 µM PdPc solution in toluene, which was used as a reference for the comparative ϕ_{UC} determination.

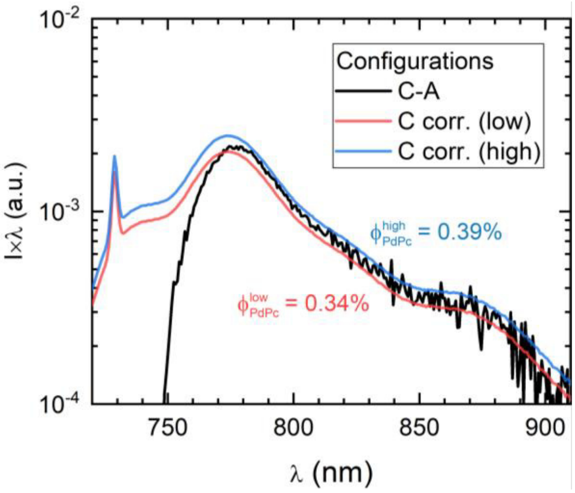

Fig. S11 Acquisition of the ϕ_{PdPc} value range, achieved by varying the scaling factor of C_{corr} spectrum.

Upconversion quantum yield (ϕ_{UC}).

From Eq. (S3), and assuming the dominant uncertainty arises from the reference yield ϕ_{PdPc} (while spectral integrals I_{UC} and I_{PdPc} contribute negligibly due to high signal-to-background; Fig. S4), we estimate:

$$\Delta\phi_{UC} \approx \frac{\Delta\phi_{PdPc}}{\phi_{PdPc}}\phi_{UC}, \qquad (S7)$$

which gives a constant relative uncertainty $\frac{\Delta\phi_{UC}}{\phi_{UC}} \approx 0.11$ for all solutions.

Asymptotic UC quantum yield (ϕ_{UC}^{∞}).

ϕ_{UC}^{∞} was obtained by fitting (Fig. S5). The relative fit error $\left(\frac{\Delta\phi_{UC}^{\infty}}{\phi_{UC}^{\infty}}\right)_{fit}$ is found to be small (0.001–0.025) compared to the propagated uncertainty from ϕ_{UC} ($\frac{\Delta\phi_{UC}}{\phi_{UC}} \approx 0.11$), hence:

$$\Delta\phi_{UC}^{\infty} \approx \frac{\Delta\phi_{UC}}{\phi_{UC}}\phi_{UC}^{\infty} \approx \frac{\Delta\phi_{PdPc}}{\phi_{PdPc}}\phi_{UC}^{\infty}. \qquad (S8)$$

Spin-statistical factor (f).

Finally, Δf was obtained by propagating uncertainties in ϕ_{UC}^{∞}, ϕ_{FL}, and ϕ_{TET}:

$$\Delta f = f\sqrt{\left(\frac{\Delta\phi_{UC}^{\infty}}{\phi_{UC}^{\infty}}\right)^2 + \left(\frac{\Delta\phi_{TET}}{\phi_{TET}}\right)^2 + \left(\frac{\Delta\phi_{FL}}{\phi_{FL}}\right)^2}. \qquad (S9)$$

All resulting uncertainties are summarized in Table S4 and are reflected directly as the error bars in Fig. 5, thereby quantifying the range of f values supported by the measurements.

Table S4. Propagated uncertainties for all experimentally determined and derived photophysical parameters used in the error analysis, as a function of TES-ADT concentration.

$c_{TES-ADT}$ (mM)	$\Delta\tau_{PdPc}$ (ns)	$\Delta\phi_{TET}$ (%)	$\Delta\phi_{FL}$ (%)	$\Delta\phi_{UC}$ (%)	$\Delta\phi_{UC}^{\infty}$ (%)	Δf (%)
0.5	34	6.72	6.65	0.10	0.16	10.14
1	13	5.41	4.14	0.15	0.19	2.91
2.5	7	3.40	4.29	0.38	0.62	4.28
5	5	2.10	2.75	0.74	0.87	4.37
10	3	1.30	2.96	0.47	0.54	2.80
20	2	0.76	4.28	0.51	0.63	6.78
40	1	0.40	1.56	0.38	0.50	7.02
80	1	0.22	1.05	0.22	0.29	8.92
UC film	5	0.46	0.23	0.04	0.06	10.60

Impact of triplet recycling on the spin-statistical factor

Back-FRET not only lowers the observed annihilator fluorescence yield (ϕ_{FL}) but can also recycle excitations by generating new sensitizer triplets, potentially biasing the inferred f. To

quantify this, we introduce a simple steady-state recycling model for the TTA-dominated regime ($\phi_{\mathrm{TTA}} \approx 1$). Without recycling, the standard relation is

$$\phi_{UC}^{\infty} = \frac{1}{2}\phi_{ISC}\phi_{TET} f \phi_{FL}. \tag{S10}$$

Including recycling, each loop contributes a factor

$$\alpha = \frac{1}{2}\phi_{ISC}\phi_{TET} f \phi_{bFRET}, \tag{S11}$$

meaning that a fraction α of the triplet population is regenerated in each successive back-FRET/ISC/TET cycle. Consequently, the effective triplet yield equals the sum of the initial and recycled contributions, $r_{\mathrm{eff}} = r_0(1 + \alpha + \alpha^2 + \cdots) = r_0/(1-\alpha)$ (for $|\alpha| < 1$). Therefore, the effective triplet yield is multiplied by $1/(1-\alpha)$, giving

$$\phi_{UC,eff}^{\infty} = \frac{\phi_{ISC}\phi_{TET} f \phi_{FL}}{2 - f\phi_{ISC}\phi_{TET}\phi_{bFRET}}. \tag{S12}$$

Solving for the intrinsic f (denoted f_i) yields

$$f_i = \frac{2\phi_{UC,eff}^{\infty}}{\phi_{ISC}\phi_{TET}\left(\phi_{FL} + \phi_{bFRET}\phi_{UC,eff}^{\infty}\right)}. \tag{S13}$$

To estimate ϕ_{bFRET} under the most demanding solid-state conditions, where back-FRET is strongest, we prepared neat TES-ADT films and films with 0.1 wt% PdPc under identical spin-coating conditions. The FL quantum yield was found to decrease from 3.20% to 2.17%, giving

$$\phi_{bFRET} = 1 - \phi_{FL}^{wPdPc}/\phi_{FL} = 0.322. \tag{S14}$$

Using the film parameters in the manuscript ($\phi_{\mathrm{UC,eff}}^{\infty} = 0.004$, $\phi_{\mathrm{FL}} = 0.016$, $\phi_{\mathrm{ISC}} = 1$, $\phi_{\mathrm{TET}} = 0.944$) yields $f_i = 49.0\%$, compared to $f = (53.0 \pm 10.6)\%$ from Eq. S10 without recycling. Thus, even in this worst-case scenario, triplet recycling via back-FRET changes f by only ~4 percentage points. As this is well within our experimental uncertainty, it does not affect the conclusions.

References

1 E. Radiunas, S. Raišys, S. Juršėnas, A. Jozeliūnaitė, T. Javorskis, U. Šinkevičiūtė, E. Orentas and K. Kazlauskas, *J. Mater. Chem. C*, 2020, **8**, 5525–5534.

2 Y. Murakami and K. Kamada, *Phys. Chem. Chem. Phys.*, 2021, **23**, 18268–18282.
